# A comparative study of loss functions and attention mechanisms in landslide semantic segmentation using U-Net

**DOI:** 10.1038/s41598-025-31789-2

**Published:** 2026-01-07

**Authors:** Vibha B. Hegde, S. Girisha, Dasharathraj K. Shetty, P. Sughosh, Balakrishna S. Maddodi, G. Savitha, Jayaraj Mymbilly Balakrishnan

**Affiliations:** 1 Manipal Institute of Technology, Manipal Academy of Higher Education, Manipal, India; 2 Department of Civil Engineering, Manipal Institute of Technology, Manipal Academy of Higher Education, Manipal, India; 3 Department of Emergency Medicine, Kasturba Medical College, Manipal Academy of Higher Education, Manipal, India

**Keywords:** Attention mechanism, Digital elevation models, GradCAM, Landslide detection, Semantic segmentation, U-Net++, Environmental sciences, Natural hazards

## Abstract

Advancement in landslide detection can be largely attributed to the introduction of deep learning, particularly semantic segmentation. Susceptible regions can be identified using satellite imagery and Digital Elevation Model (DEM) data. This study explores multi-modal data to improve the identification and detection of landslides. The U-Net model serves as a baseline that is further enhanced by the introduction of an attention mechanism that refines pixel-level predictions. Evaluation of various loss functions resulted in increased performance optimization. The Bijie landslide dataset, featuring high-resolution satellite images, DEM data, and ground truth masks, was used for training and evaluation. Precision, recall, F1 score, accuracy, mean intersection over union (mIoU), and Area Under the Curve (AUC) were metrics used to evaluate the performance. The model incorporating the attention mechanism achieved the highest mIoU of 0.76, F1 score of 0.74, and accuracy of 0.94, surpassing the base model. Attention mechanisms concentrate on critical regions and thus improve feature extraction by enhancing segmentation precision. The integration of multi-modal data and diverse loss functions contributes to better landslide detection.

## Introduction

A landslide is the downward movement of rock, debris, or the earth along a slope. There are multiple causes for landslides. Landslides can be triggered by heavy rainfall, soil erosion, rising groundwater levels, earthquakes, snowmelt, volcanic activity, or human activities that result in rising temperatures and climate change. Effective management of landslides is difficult due to limited resources, data availability, and technical expertise, as mentioned by Varol et al.^1^. A study by Ćmielewski et al.^2^ highlights that field surveys and manual remote sensing data interpretation have traditionally been the main methods for landslide detection. These methods are time-consuming, labor-intensive, and expensive. They also relied on expert knowledge and weather changes. Yamada et al.^3^ addresses the automatic and precise detection of landslides using satellite or aerial images. Tracking and detecting landslides in real-time can reduce the risk by providing quick rescue operations and reducing the possible loss of life and property. A systematic study by Mishra et al.^4^ focuses on the development of remote sensing with special emphasis on landslide detection and its methodologies. Ground-based remote sensing techniques have grown significantly, owing to their improved data quality, technological advancements, fast processing and measurement times, reduced costs, and enhanced accuracy. Remote sensing is useful for the detection, mapping, and monitoring of landslides during emergencies and post-disasters. Vasanthkumar wrote on the applications of remote sensing and observation, particularly on the popular imaging method known as Synthetic Aperture Radar (SAR), in his study Vasanthkumar et al.^5^. It allows for detailed imaging of extensive areas of land, delivering high-quality images even during harsh weather conditions, both at night and during the day. A study by Salleh et al.^6^ supports the idea that remote sensing technology can be used to detect vegetation irregularities, which can then act as biological evidence for potential landslide occurrences. This method provides new insights for landslide mapping. Artificial Intelligence (AI) is being utilized in remote sensing to help with tasks such as image processing, identification, and recognition as highlighted in the studies by Ouchra et al.^7^, Ouchra et al.^8^, Lai et al.^9^. This makes it possible to swiftly locate objects in satellite imagery. Satellite image classification converts satellite imagery into useful information. In a study by Casagli et al.^10^, ground-based remote sensing methods, such as Laser Detection and Ranging (LiDAR), Doppler Radar, and Ground-Based Interferometric Radar, are being utilized more often for the early detection of landslides and real-time monitoring. LiDAR, an active remote sensing method was also used in a study by Theriault et al.^11^. It uses electromagnetic energy to identify objects, measure distances, and infer physical characteristics from the radiation interactions. LiDAR detection analysis compares successive datasets to detect elevation changes, aiding the assessment of landslide activity. As reported by Saini et al.^12^, satellite remote sensing has emerged as an important tool for researching and monitoring landslides across areas, with potential applications in hazard assessment and disaster response. Machine learning techniques, object-based image analysis, and the integration of multiple sensors have improved the accuracy of landslide detection. Digital photogrammetry and satellite InSAR are two examples of multi-source remote sensing technologies that have been effectively utilized for analyzing landslide characteristics in Choi et al.^13^. Yang et al.^14^ discusses the effective feature extraction capabilities of Convolutional Neural Networks (CNNs), allowing the automatic selection of relevant landslide features, reduced time consumption, and improved accuracy in landslide susceptibility assessment. The ResUNet model was used, which integrates spatial and channel attention modules with a transformer to improve landslide detection using small datasets. This achieved the highest mIoU and F1 scores, outperforming regular ResUNet. A multi-dimensional CNN coupling model that connects 1D-CNN and 2D-CNN was proposed by Zhao et al.^15^. The model uses asymmetric aggregation to improve landslide susceptibility assessment by reducing the computational complexity and overfitting. The method used in this paper presents a U-Net++ architecture reinforced with Gradient-weighted class activation mapping (GradCAM) and attention mechanisms to detect landslide-prone areas from satellite imagery and Digital Elevation Model (DEM) data. It captures high-level semantic features and fine-grained spatial details. The interpretability of the model was improved by integrating GradCAM, which provides visual explanations for its predictions. Different loss functions were explored to assess how well the performance of the model was optimized.

## Related work

CNNs have become a cutting-edge technology in computer vision for landslide detection. This section outlines the methods and techniques currently employed for landslide detection and segmentation.

### Advancements in CNNs for landslide detection

Few studies such as Yi et al.^16^ have explored the transferability of CNN models trained on data from specific events to detect landslides, demonstrating their high accuracy and potential for widespread use in landslide detection. LandsNet, an end-to-end cascaded deep learning network was built to discover various landslide features. Comparative studies have highlighted the robustness and feasibility of this method, demonstrating its potential for emergency response in natural disasters. A study by Zhang et al.^17^ used three deep learning models:- CNN-12, ResNet18, and ResNet50. It focuses on the ability of CNN and deep residual networks (ResNet) to recognize landslide threats. The ResNet18 model performed better than other models in terms of recognition accuracy and other metrics. This model is not only efficient but also provides a more detailed comparison of the strengths and weaknesses of different neural network systems. A study by Cai et al.^18^ focuses on the problem of inefficient and undersampled landslide data. Densely linked convolutional networks (DenseNets) are used, which show promising applicability for detecting landslides over large regions. Liu et al.^19^ developed a novel model for automatic landslide detection from high-resolution satellite images using a Dual-Branch Contextual-Aware network (DCA-Net). It incorporates an asymmetrical encoder-decoder framework and depthwise blocks with two channels. To further improve the performance and receptive field, the model fuses two parallel channels using asymmetric depthwise separable convolutions with residual connections. A comprehensive analysis of deep learning for landslide detection was conducted by Zhang et al.^20^. Tasks were classified into various frameworks, including detection, classification, segmentation, sequence, and hybrid models- each designed for particular landslide-related applications. Few models have shown powerful capabilities in landslide detection and mapping while others are effective in creating susceptibility maps, that predict areas at risk of landslides, aiding in proactive risk management. It is challenging to swiftly deploy CNNs and other deep learning models after a landslide occurs since their efficacy depends on the quality and availability of training data. A study by Asadi et al.^21^ presents a model for regional landslide mapping that was developed using a deep transfer learning framework. The model was based on DeepLabV3+ and demonstrated high accuracy in identifying landslide features without requiring particular model adjustments or training data for each event. A study by Sameen et al.^22^ discusses how deep learning methods, such as CNNs, enhance landslide mapping outcomes in comparison to traditional machine learning. However, the performance of CNN models differs based on the input data, underscoring the importance of selecting appropriate network architectures and data fusion techniques for optimal results. Residual networks exhibit better generalization and faster convergence for testing, focusing on the consequences of architectural choices on performance. Research conducted by Pargaonkar et al.^23^ brings to the fore the importance of U-Net architecture, particularly for semantic segmentation tasks. The models included U-Net and ResUNet architecture. The ResUNet model was the most effective. A paper by Ruilong Wei et al.^24^ discusses an Attention and Multiscale mechanism with the U-Net (AMU-Net) model for attention-based feature enhancement in landslide detection. A skip connection with attention mechanism was added to the convolution layer, to adopt a multiscale technique. Lin et al.^25^ suggest that modifying the U-Net model by adding a Convolutional Block Attention Module (CBAM-U-net) leads to a performance enhancement of the model. They have been proven to significantly improve the capacity of feature learning, representation, adaptation, and refinement leading to improved accuracy and robustness in the detection and classification of high-precision landslide images. The other comparative models that were considered include DeepLabv3+, U-Net, and a Fully Convolutional Network (FCN). The results showed that CBAM-U-Net outperformed the other models in terms of performance. Researchers Dong et al.^26^proposed a new design called L-Unet that integrates a Multiscale Feature Fusion (MFF) module, a residual attention network, and high-level advanced upsampling techniques. This model could accurately and effectively identify landslides, exceeding the standard U-Net model.

### The utilization of hybrid models and data augmentation

The limited number of landslide samples available for training purposes poses difficulties for models tailored for landslide detection. Jiang et al.^27^ sought to improve landslide detection by using a Mask Region based CNN (Mask R-CNN) model. The researchers created replicated hard samples by modifying the forms, colors, and textures of available landslide samples and adding more complex backgrounds. This approach aims to boost the performance of the model against false positives. The Mask R-CNN model was trained using both real and simulated hard samples. In this study, it was reported that the Mask R-CNN model produced with simulated hard samples improves the accuracy using a few data sets. Chen et al.^28^ have also reported that CNN-based models for landslide detection have constraints, such as class complexity within the topography, class imbalance due to underrepresentation of training data, and high computational costs. This research explores a different method for landslide detection using multi-channel optical remote sensing data, which is of great importance in disaster management and mitigation. To enhance the detection accuracy, a hybrid architecture known as the Conv-Trans Dual Network (CTDNet) is used, which integrates the advantages of CNNs and Swin transformers.

### Integration of multi-modal and multiscale data

In a study by Tanatipuknon et al.^29^, machine learning models have been recommended to enhance detection accuracy. These models performed considerably better when used with cross-modal data. The model uses RGB images, and DEMs accompanied by decision tree classification, which improves the accuracy, precision, recall, and F-measure when compared to other methods. A deep learning model based on a Fully Convolutional Spectral-Topographic Fusion network (FSTF-Net) was developed in another study by Xia et al.^30^. To enhance the performance, this method employs a deep CNN that integrates information from various sources, including topographical elements. The model demonstrated notable enhancements in both the overall classification accuracy and the precision of landslide detection. A study by Piralilou et al.^31^ discusses the use of multiscale segmentation and classification techniques in conjunction with DEM data to improve the identification of landslides by considering different scales and temporal changes. This study detected landslides in the upper Himalayas by combining different machine learning models with multiscale image segmentation. This has been shown to significantly improve the overall detection accuracy.

## Dataset

**Fig. 1:**
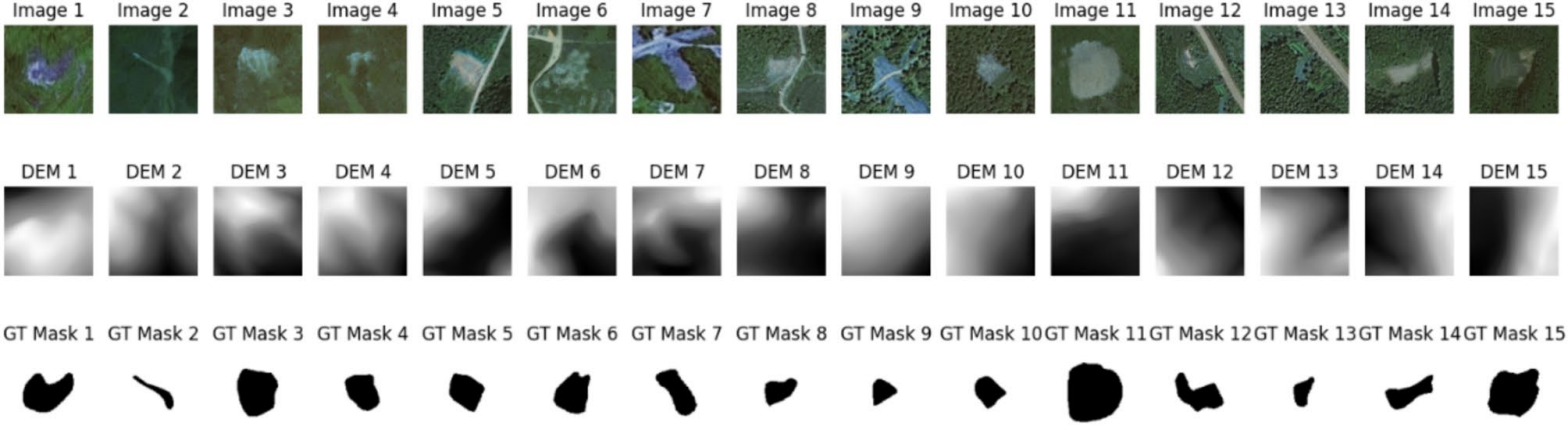
Satellite images, corresponding DEMs, and ground truth masks.

The dataset was publicly accessible. It was gathered from the Bijie region of Guizhou Province, China. The terrain complexity and the surrounding environment make this area extremely prone to landslides. This is valuable as it includes a diverse set of landslides, including rockfalls, debris slides, and rock slides, along with corresponding non-landslide data. It consists of 770 satellite images, that are essential for locating and segmenting landslide areas. The DEM data, which provide topographic information, were also included in the dataset. DEM data are crucial for precise landslide identification and segmentation, as they help in understanding the characteristics of terrain features, such as aspect, slope, and elevation. It is the grayscale depiction of the terrain’s elevation, with darker shades denoting lower elevations and lighter shades denoting higher elevations, as mentioned by Shunping Ji. et al.^32^. The DEM data was paired with 2D high-resolution remote sensing images to enhance the feature extraction process and facilitate better landslide segmentation as it offers more terrain context. To train and assess segmentation models in landslide detection tasks, this dataset additionally contains ground truth masks that categorize each pixel as landslide or non-landslide. Hence, this dataset is useful for semantic segmentation, because it integrates multiple multi-modal data sources. This dataset effectively represents both landslide-prone and non-landslide areas, and hence is well-balanced. This eliminated the concerns of data imbalance during model training and evaluation. Figure 1 shows a few landslide sample images, the associated DEM, and the ground truth mask.

### Ethics statement

The study used publicly available data from http://gpcv.whu.edu.cn/data/Bijie_pages.html, which is an open-source online repository. The data was accessed and used in compliance with the terms and conditions outlined by the database provider.

## Methods

In this study, U-Net architecture was implemented as the base model to identify landslide regions. U-Net served as the base model for several reasons. First, the encoder-decoder structure of U-Net enables effective feature extraction and fusion. Skip connections aid in the retention of spatial information. This approach leverages the strength of U-Net for image segmentation and enhances it by integrating data to process both satellite and elevation data.

### U-Net architecture

**Fig. 2:**
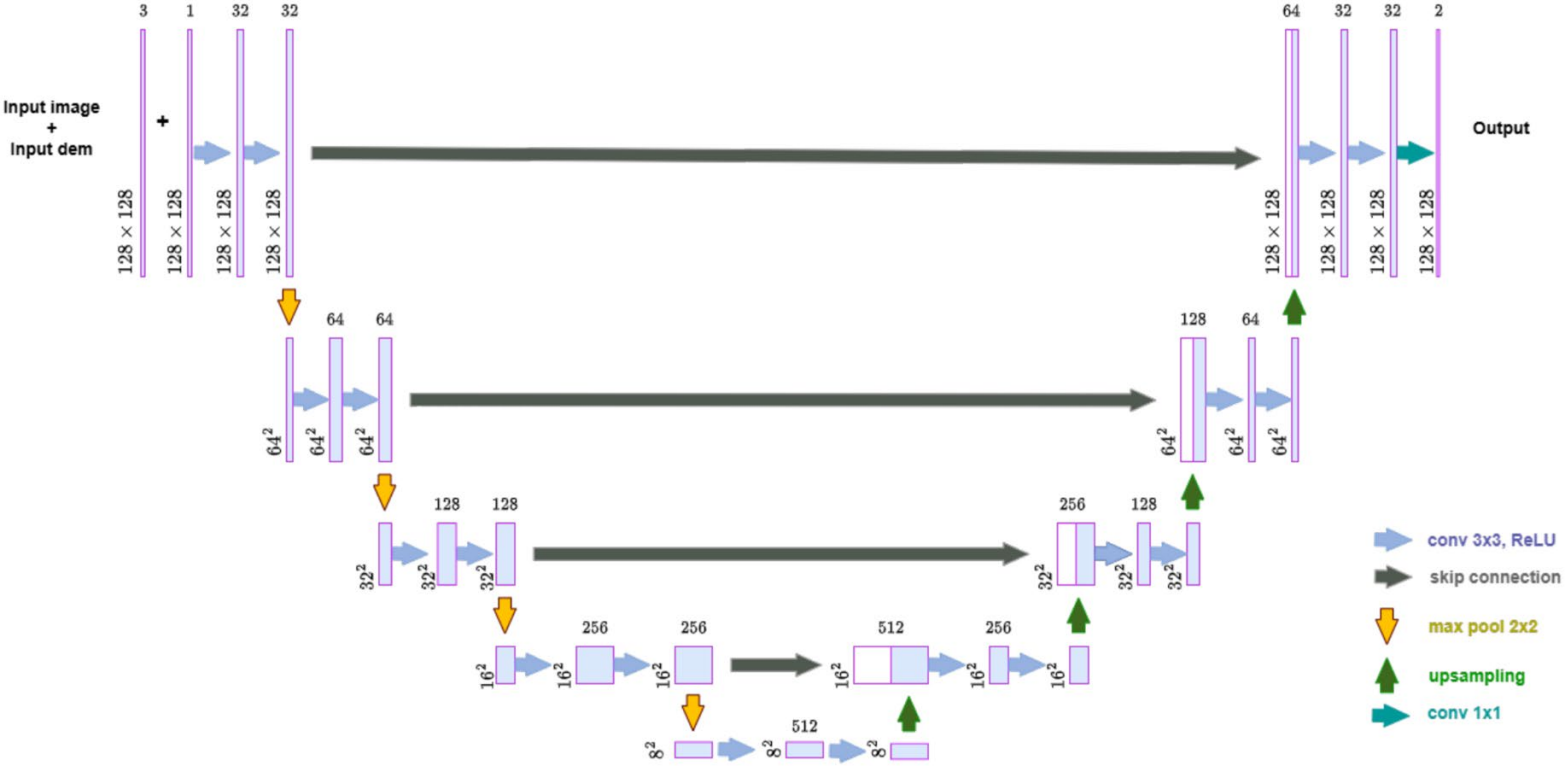
U-Net architecture.^35^.

U-Net is a type of CNN originally intended for the segmentation of biological images but has been adapted for other applications, such as landslide detection. It features an encoder-decoder structure that captures both local and global features through its unique U-shaped design. The encoder captures the context by progressively compressing the spatial dimensions of the feature maps while increasing the depth. The segmentation map is constructed in finer detail by the decoder, using the upsampling layers that restore the spatial dimensions alongside the features received from the encoder with the use of skip connections, Salim et al.^33^. Skip connections between the layers of encoder and decoder preserve spatial information, Lu et al.^34^. The U-Net architecture for landslide detection leveraged highly precise satellite images to correlate with high-precision DEM data. Satellite images provided high-context information about the surface and terrain features. The elevation changes, slopes, and other aspects, which are collectively termed topography, are provided by DEMs. DEMs are crucial for understanding the likelihood that the terrain is prone to landslides. In addition, U-Net is further enhanced by stacking the image and DEM input. The stacked input is processed using a single encoder. This makes the model better equipped to capture diverse features from various data sources. Therefore, the model can capture both low-level spatial detail and high-level semantic data. The encoder path is tasked with reducing the resolution of the input image to identify the features at various scales. This segment of the network identifies the high-level semantic features that are crucial for landslide detection. It includes several convolutional layers, activation functions, and pooling layers. The satellite images that are fed to the encoder focus on global structures, such as wide regions that are susceptible to landslides. The DEM data emphasize detailed and localized data, including slight changes in texture, slope, or elevation, which can reveal regions at risk for landslides. The decoder path is in charge of rebuilding the spatial dimensions of the input image from the features extracted by the encoder. Figure 2 shows the U-Net architecture.

**Fig. 3:**
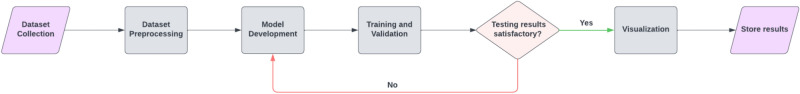
The workflow diagram.

In this study, several landslide detection models were implemented using the U-Net framework. They maintain a consistent core structure, but exhibit variations in their loss functions, slight differences in the design of the encoder and decoder components, and attention mechanisms. A workflow diagram showing each step is shown in Figure 3.

### Model 1: U-Net

A standard U-Net architecture for landslide detection uses stacked inputs for satellite imagery and DEM data. Pixel-wise semantic segmentation was performed to detect landslides in the input images. The encoder extracts features from both inputs (satellite images and DEM), after which the feature maps are concatenated in the bottleneck. Combining these features enables the network to integrate spatial and topographic data, which are essential for identifying intricate patterns indicative of landslides. The decoder rebuilds the high-resolution segmentation map by upsampling the feature maps.

### Model 2: ResUNet

The architecture resembles U-Net but is enhanced with residual blocks. Residual blocks were integrated into the architecture to mitigate the vanishing gradient problem, which is a prevalent issue in deep neural networks. These blocks simplified the learning process. It uses skip or shortcut connections, which add input to the output by avoiding single or multiple layers. This allows gradients to move through the network more easily, thus making training deep networks more feasible and efficient, Shen Z. et al.^36^.

### Model 3: U-Net++

U-Net++ is a sophisticated deep-learning framework aimed at enhancing the effectiveness of the original U-Net architecture. As reported by Li B. et al.^37^, U-Net++ employs dense skip connections consisting of stacked convoluted blocks. These blocks were implemented within the skip connections. U-Net++ enhances the segmentation quality of images and feature representation by using indent convolutions and dense skip connections.

### Model 4: U-Net++ with spatial attention

U-Net++ uses spatial attention features to assess the importance of different spatial locations in the feature maps, as a way of helping the network pay more attention to key places. It focuses on the spatial connections that are relevant to the image which enhances the model’s ability to discriminate between different areas based on their spatial contexts, Lu C. et al.^38^. Spatial attention was implemented by applying average and max pooling along the channel axis. This was implemented following each convolution operation within the encoder blocks.

### Model 5: U-Net++ with contextual attention

The contextual attention mechanism increases the focus of the model on relevant features by adjusting the significance of different parts and channels in the input data. This mechanism captures a larger context of the image which is important for understanding the complete scene and making better segmentation decisions, Jiang J. et al.^39^. The Contextual attention module integrates both channel and spatial attention. It starts with channel attention, utilizing global average pooling and max pooling to create descriptors that emphasize significant feature channels. This is followed by spatial attention, which integrates pooled spatial statistics and refines spatially significant regions.

### Model 6: U-Net++ with spatial and contextual attention

U-Net++ incorporates both spatial and contextual attention. The integration of spatial and contextual attention mechanisms helps in improved feature extraction and capturing finer details and broader context, Liu M. et al.^40^ This combination was incorporated sequentially following each convolutional and batch normalization layer in the encoder pathway. While prior works have utilized single attention mechanisms like CBAM or spatial attention alone, this study combines both spatial and contextual attention modules. Spatial attention enhances local pixel-level details, whereas contextual attention identifies global feature dependencies.

### Parameter optimization

#### Loss function

Binary Cross-Entropy (BCE) has been utilized in binary classification tasks. It calculates the discrepancy between predicted probabilities and the true class labels, Usha et al.^41^. Focal loss modifies the standard cross-entropy loss by increasing the loss for hard examples while keeping it low for easy ones, Yang et al.^42^. Dice loss evaluates the overlap between the ground truth and predicted segmentation. Further, Dice loss is robust to small isolated regions^43^. The Weighted Categorical Cross-Entropy (WCE) assigns different weights to different classes. By extending better importance to underrepresented classes, it aids the model’s ability to detect smaller and less prominent instances, Huang et al.^44^

Equation (1) defines binary cross-entropy loss:

1 $$\begin{aligned} L_{BCE} = -\frac{1}{X} \sum _{i=1}^{X} \left[ y_i \log (v_i) + (1-y_i) \log (1-v_i) \right] \end{aligned}$$

where $X$ is the number of samples, $y_i$ is the actual label (0 or 1), and $v_i$ is the predicted probability.

The focal loss is given by Eq. (2):

2 $$\begin{aligned} L_{FL} = -a(1-v_t)^b \log (v_t) \end{aligned}$$

where $v_t = y v + (1 - y)(1 - v)$ is the predicted probability for the actual class, $a$ is the balancing factor, $b$ is the focusing parameter, $v$ is the predicted probability, and $y$ is the actual label (0 or 1).

The dice loss is given by Eq. (3):

3 $$\begin{aligned} L_{\textrm{Dice}} = 1 - \frac{2\sum _{i=1}^{X} y_i v_i}{\sum _{i=1}^{X} y_i + \sum _{i=1}^{X} v_i} \end{aligned}$$

where $y_i$ is the actual label, $v_i$ is the predicted output, and $X$ is the number of samples.

The weighted categorical cross-entropy loss is given by Eq. (4):

4 $$\begin{aligned} L_{\textrm{WCE}} = - \frac{1}{X} \sum _{i=1}^{X} \sum _{c=1}^{C} w_c y_{i,c} \log (v_{i,c}) \end{aligned}$$

where $C$ is the number of classes, $X$ is the number of samples, $y_{i,c}$ is the actual label for samples $i$ and class $c$, $v_{i,c}$ is the predicted probability for samples $i$ and class $c$, and $w_c$ is the weight of class $c$.

#### Optimizer and learning rate

These are critical components of deep learning. This significantly affects the training and performance of the neural networks. An optimizer minimizes the loss function by altering the parameters of the network, thereby improving the accuracy and efficiency of the model. The learning rate adjusts the step size at each iteration. It is crucial as it affects how quickly or slowly a model learns, Hassan et al.^45^. The Adam optimizer was chosen for its adaptive learning rate and efficient gradient-based optimization. A learning rate of 0.00001 was used in training. This learning rate provided a good balance between convergence speed and performance stability.

#### Dropout

This regularization method is used in training neural networks to avoid overfitting, which occurs when a model performs poorly on unknown data after learning the training data too well. The key idea behind dropout is to randomly drop units (neurons) and their connections during the training process. The dropout rate was 0.5.

#### Batch normalization

Batch normalization is a widely used technique that normalizes the activation of intermediate layers within mini-batches. It transforms the activation function to improve the training and performance of models. Batch normalization was applied after each convolutional block in order to accelerate training and stabilize learning.

#### Batch size and epoch

One of the crucial hyperparameters is the batch size, which is used particularly for training neural networks. It describes the number of training examples used in a single iteration of model training. The training dynamics of a model are influenced by the batch size. Smaller batch sizes often lead to better generalization but may result in longer training times owing to more frequent updates. Conversely, larger batch sizes can speed up training but may suffer from poorer generalization. The batch size was set to 2. We achieved successful convergence despite the small batch size. Each model was trained for 50 epochs, as this allowed sufficient time for learning without overfitting.

## Experiments and analysis

### Experimental setup

Experiments were conducted using Google Colab. The hardware accelerator used in this setup is a T4 GPU, which significantly improves the performance of tasks that require intensive computations. Colab uses a Virtual Machine (VM) environment to run Python code. Additionally, data can be easily saved and loaded from Google Drive, providing easy storage and accessibility.

### Model training

The training set for each model comprised of 80% of the data, within which 20% was used for validation and the remaining 20% was set aside for testing. The Bijie landslide dataset, consisting of high-resolution satellite images and the corresponding DEM data, was used. To standardize the input dimensions, the input images were resized to 128 x 128 pixels. The DEM data was resampled to match this resolution and normalized to ensure consistency with the scale of the satellite image inputs, which were also normalized. The DEM data were stacked with satellite images, forming a single combined input to maintain spatial alignment and synchronization. This method represents an early fusion strategy that allows the model to learn spatial and elevation features right from the start of the learning process. The three base models were trained using four loss functions. Twelve preliminary experiments were conducted using four loss functions. The training time for the model ranged from 5 to 9 min. The primary criterion for selecting the best loss function for each model was the mIoU. The significance of using the mIoU is to evaluate the model’s potential to predict both minority and majority classes. The three base models were further evaluated by using the loss function that achieved the highest mIoU score. To build on these results, attention mechanisms were incorporated into the top-performing base model that achieved the highest mIoU score. Specifically, three different types of attention mechanisms are incorporated: spatial attention, contextual attention, and a combination of spatial and contextual attention. After introducing the attention mechanism, the average training time per epoch increased from 5 sec to 10 sec. The inference time per image increased from 3–4 ms to 4–5 ms. The process of training U-Net++ using an integrated attention mechanism is outlined in Algorithm 1.

**Algorithm 1:**
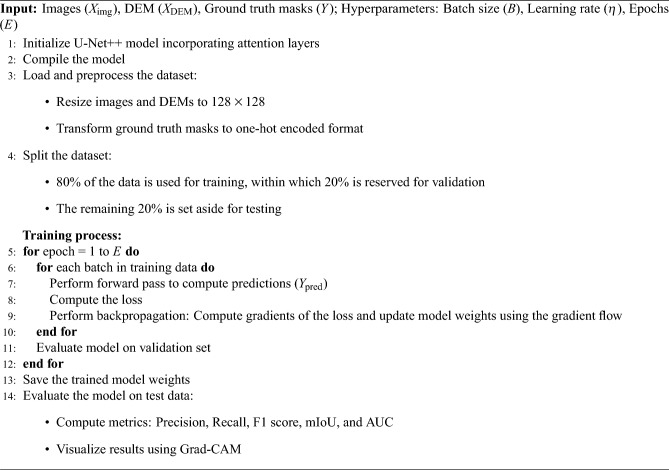
Training process for U-Net++ with attention mechanism.

## Results

Twelve preliminary experiments were conducted using four different loss functions, with the best three selected based on their performance as shown in Table 1.

**Table 1:** Comparison of mIoU scores for base models using various loss functions. Bold numbers indicate the best results.

Model	BCE	Focal loss	Dice loss	WCE
U-Net	**0.575**	0.572	0.557	0.508
ResUNet	0.770	0.786	0.776	**0.787**
U-Net++	0.770	0.766	**0.774**	0.759

**Table 2:** Performance metrics of Base models. Bold numbers indicate the best results.

Model	mIoU	AUC Score	Precision	Recall	F1 Score	Accuracy
U-Net	0.50	0.80	0.94	0.12	0.21	0.90
ResUNet	0.70	0.94	0.85	0.53	0.65	0.94
U-Net++	**0.75**	0.87	0.69	0.77	0.73	0.94

### A comparison of different loss functions

The three base models were further evaluated using the loss function that attained the highest mIoU score. These models were assessed using a few metrics, and the results for each model are listed in Table 2.

#### U-Net

A simple U-Net architecture with a BCE loss function is used in this model. BCE is widely used for binary classification and it works well for simpler architectures, such as U-Net. However, for the landslide semantic segmentation task, this model obtained the lowest mIoU value of 0.50. Hence, BCE is not suitable for landslide semantic segmentation.

#### ResUNet

This model uses a residual block with WCE loss function. Generally, the WCE learns both classes (landslide and non-landslide) thoroughly. However, they may not effectively handle small-scale landslides. The mIoU score obtained for this model was 0.70, which was higher than that of the U-Net.

#### U-Net++

U-Net++ incorporates nested and dense skip connections, which help in better feature propagation and gradient flow. This model uses the Dice loss function. Dice loss focuses on the overlap between the ground truth and the predicted segmentation, which helps accurately delineate the boundaries of landslides. The mIoU score obtained for this model is 0.75 and is higher than that of both U-Net and ResUNet. Dice Loss can better handle small-scale landslides by ensuring that even small regions are accurately segmented, which is a significant challenge for the BCE and WCE. Hence, the Dice loss function is a significantly better loss function for landslide semantic segmentation.

In a further analysis, attention mechanisms were integrated into the leading base model- U-Net++, as it attained the highest mIoU score of 0.75.

**Fig. 4:**
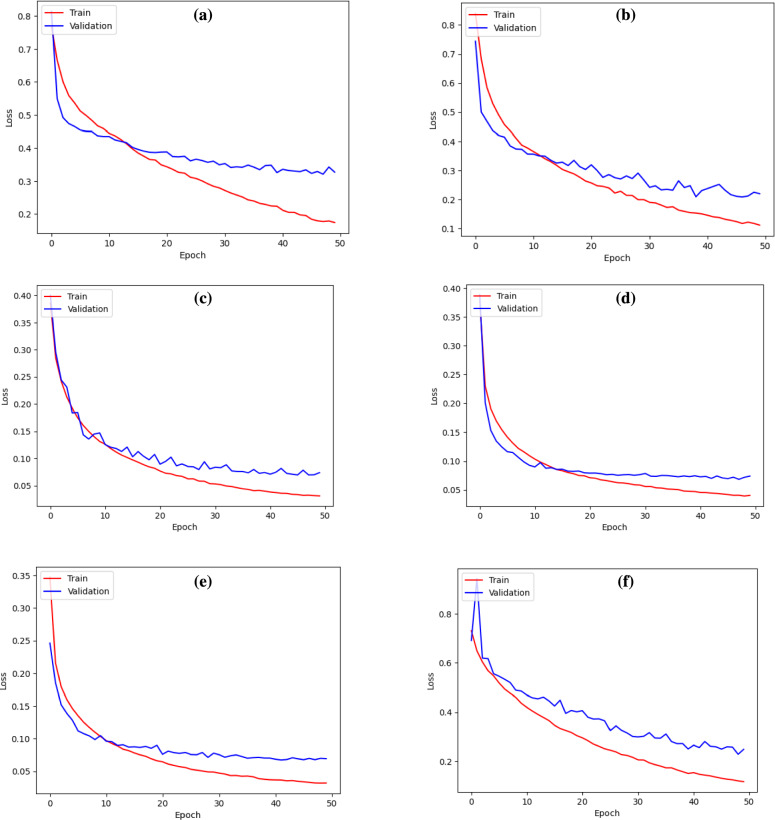
Loss curves for different models across training and validation. (**a**) U-Net, (**b**) ResUNet, (**c**) U-Net++, (**d**) U-Net++ (spatial attention), (**e**) U-Net++ (contextual attention), (**f**) U-Net++ (spatial+contextual attention).

**Fig. 5:**
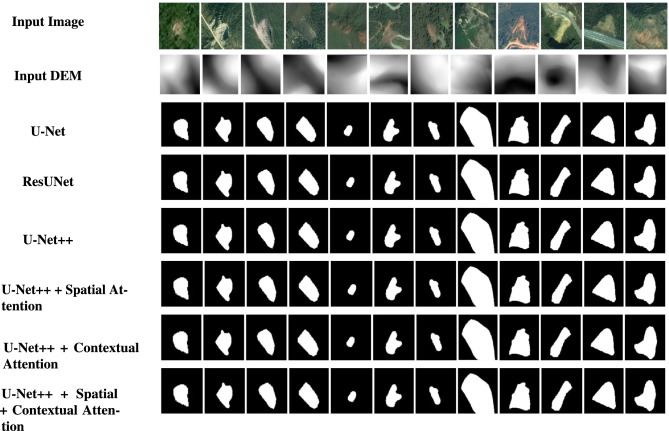
Test image samples and the corresponding predictions for each model.

### A comparison of base models and attention mechanism models

Each of the base models and attention-based models was evaluated to determine their impact on performance metrics, with particular emphasis on mIoU and a few other metrics, as shown in Table 3.

#### U-Net

The U-Net model, which was trained using BCE achieved a precision score of 0.94, indicating that the model performed well in identifying landslide regions without many false positives. However, it faced a few challenges as it had a low recall of 0.12 for the landslide class, indicating that the model was not very effective in identifying true positives. This has resulted in many landslides being missed. This issue is also further evident by the model’s low F1 score of 0.21 and an mIoU of 0.5. The AUC score of 0.8 shows its limited ability to differentiate between landslide and non-landslide areas.

#### ResUNet

The ResUNet model, which was trained using WCE, outperformed U-Net, as evidenced by its higher recall of 0.53 and F1 score of 0.65. The model’s mIoU of 0.7 significantly surpassed that of U-Net, indicating an enhanced capability in segmenting landslide regions. ResUNet achieved high AUC of 0.94 as it generalizes well for broad area classification. However, it may be less effective in capturing the fine structure of landslides due to the absence of dedicated attention mechanisms that refine local and contextual details.

#### U-Net++

U-Net++, which uses Dice loss, achieved a good mIoU score of 0.75. With a recall rate of 0.77, U-Net++ was good for identifying true positives. However, this came at the cost of a model precision of 0.69, which yields a higher rate of false positives compared to other models. The F1 score was 0.73 and, an AUC score of 0.87 suggests a slight decrease in classification confidence.

#### U-Net++ with spatial attention

U-Net++ incorporated with spatial attention achieves an mIoU score of 0.72. It had a precision of 0.73, demonstrating a strong capability to accurately classify landslide pixels with few false positives. However, the recall was 0.65, indicating that the model missed a few landslide pixels. This could be a problem when complete landslide detection is crucial. It has a slightly reduced F1 score of 0.69 and an AUC score of 0.82, indicating a slight compromise in classification capability.

#### U-Net++ with contextual attention

This variant of U-Net++ with contextual attention achieved the highest mIoU score of 0.76 and AUC score of 0.9. While AUC is slightly lower than ResUNet, the trade-off is minimal given the enhanced segmentation quality. It had a recall of 0.79, which is crucial in cases where detecting all possible instances of landslides is critical. Although the precision was 0.69, leading to more false positives, the F1 score of 0.74 shows an ideal balance between precision and recall. This makes this model effective for landslide detection scenarios where it is crucial to identify all potential landslide regions.

#### U-Net++ with spatial and contextual attention

The U-Net++ model with spatial and contextual attention is a balanced model that results in an mIoU score of 0.73 and an AUC score of 0.93. It has a precision of 0.73, maintaining low false positives, a recall of 0.65, and an F1 score of 0.69.

The train-validation loss curves are shown in Fig. 4. Figure 5 illustrates the test image samples and their respective predictions from each model, showing the segmentation performance across various architectures.

**Table 3:** Performance metrics of base models and attention models. Bold numbers indicate the best results.

Model	mIoU	AUC score	Precision	Recall	F1 score	Accuracy
U-Net	0.50	0.80	0.94	0.12	0.21	0.90
ResUNet	0.70	0.94	0.85	0.53	0.65	0.94
U-Net++	0.75	0.87	0.69	0.77	0.73	0.94
U-Net++ (spatial attention)	0.72	0.82	0.73	0.65	0.69	0.94
U-Net++ (contextual attention)	** 0.76**	0.90	0.69	** 0.79**	**0.74**	**0.94**
U-Net++ (spatial+contextual attention)	0.73	0.93	0.73	0.65	0.69	0.94

### Comparative analysis with specialized remote sensing approaches

To better contextualize the performance of the proposed attention-enhanced U-Net++ model, a comparative analysis with several specialized remote sensing approaches is provided in Table 4. It summarizes key performance metrics of several models alongside the proposed model’s results.

**Table 4:** Performance comparison with specialized remote sensing approaches for landslide detection.

Model	mIoU	F1 score	References
AMU-Net (feature enhancement + attention)	0.79	0.81	Ruilong et al.^24^
ResUNet with transformer + CBAM	0.78	0.87	Yang et al.^14^
LandsNet	–	0.86	Yi et al.^16^
CTDNet (ConvNeXt + swin transformer)	–	0.74	Chen et al.^28^
Proposed U-Net++ with contextual attention	0.76	0.74	–

While AMU-Net achieves a good mIoU of 0.79, the proposed approach integrates contextual attention and combines images with DEM. While the mIoU score of 0.76 for the proposed model is slightly lower, it was attained on a more geologically diverse dataset (Bijie), showcasing competitive generalization capabilities. Although transformer-based models demonstrate enhanced global context learning with mIoU of 0.78, they frequently entail higher computational costs. LandsNet model has F1 score of 0.86. The proposed model has slightly lower F1 score of 0.74, but it focuses on general applicability across various landslide conditions while utilizing a more lightweight architecture. While CTDNet shows good performance on the Landslide4Sense dataset with F1 score of 0.74, the proposed model provides a competitive and more adaptable solution by leveraging multimodal inputs, with less computational overhead, and ensuring a balance between recall and segmentation accuracy.

**Fig. 6:**
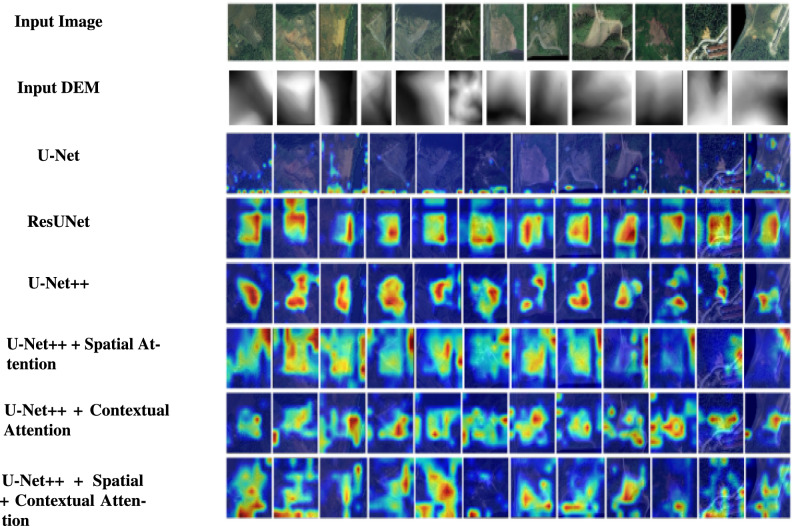
GradCAM visualization.

#### GradCAM evaluation

To make the model’s predictions easier to understand, GradCAM was incorporated along with segmentation. This feature is particularly crucial in landslide detection as it helps geologists and disaster management professionals understand which landscape areas are pivotal in the model’s predictions. GradCAMs produce visual heatmaps that identify the areas of the input image that influence the model’s decision. The landslide samples, along with the GradCAM visualizations for each model, are shown in Fig. 6.

## Contributions of sustainable development goals (SDGs)

This study on landslide detection using semantic segmentation aligns with several Sustainable Development Goals (SDGs). By utilizing deep learning and geospatial technologies, it contributes to SDG 9 (Industry, Innovation, and Infrastructure) by improving disaster prediction and infrastructure resilience. The analysis of DEM and remote sensing data facilitates early landslide identification, lowers urban risks, and builds safer communities- all of which contribute to SDG 11 (Sustainable Cities and Communities). By addressing climate-induced landslide causes, such as extreme weather and deforestation, and enhancing disaster risk reduction, this study also supports SDG 13 (Climate Action). It contributes to SDG 15 (Life on Land) by monitoring the environmental damage caused by landslides and promoting sustainable land management practices.

## Conclusion

This paper presents an advanced method for landslide detection, trained using the Bijie landslide dataset. This dataset captures a diverse set of landslide features, including high-resolution satellite images, DEM data, and ground truth masks. The models process satellite imagery and DEM data, which capture both topographical and spatial information, enabling precise segmentation. A thorough analysis of different loss functions, base models, and attention mechanism-induced models was performed to determine their effectiveness for landslide detection. A major novelty of this method is the architectural advancement of integrating both spatial and contextual attention mechanisms within the U-Net++ framework. Unlike previous studies that mainly employed single attention type, this method explicitly utilizes the complementary advantages of both spatial and contextual attention types, leading to enhanced segmentation performance. U-Net++ with Contextual Attention performed better than the other models, attaining the highest recall, F1 score, and mIoU score. The mIoU score of 0.76 achieved by this model was the highest among all other models. This model successfully integrated contextual information from the surrounding areas while concentrating on the relevant spatial regions. This feature makes the model a reliable choice for landslide detection because it enables the precise identification of areas that are prone to landslides. Through the incorporation of GradCAM visualization, the study elucidated the decision-making process of the model and emphasized the regions of the image that influenced landslide detection.

Although this study mainly relied on static images, future research may find it advantageous to incorporate temporal satellite data. This approach makes it possible to track changes in the terrain over time. The forecasting capabilities of deep learning models can be enhanced by integrating temporal and spatial data. By utilizing this combination, the models would be capable of forecasting landslides in advance, offering a proactive strategy for disaster management. Additionally, the model’s resilience and capacity to generalize across different terrains and environmental conditions can be improved by expanding the datasets and including data from a wider range of regions with diverse geological features. This would establish a stronger foundation for using these models in real-world disaster management scenarios.

## Data Availability

The dataset analyzed in the current study is available in the repository, https://www.kaggle.com/datasets/hanstankman/bijie-landslidedataset
